# Hematopoietic Stem Cell Transplantation Resolves the Immune Deficit Associated with STAT3-Dominant-Negative Hyper-IgE Syndrome

**DOI:** 10.1007/s10875-021-00971-2

**Published:** 2021-02-01

**Authors:** Stephanie C. Harrison, Christo Tsilifis, Mary A. Slatter, Zohreh Nademi, Austen Worth, Paul Veys, Mark J. Ponsford, Stephen Jolles, Waleed Al-Herz, Terence Flood, Andrew J. Cant, Rainer Doffinger, Gabriela Barcenas-Morales, Ben Carpenter, Rachael Hough, Ásgeir Haraldsson, Jennifer Heimall, Bodo Grimbacher, Mario Abinun, Andrew R. Gennery

**Affiliations:** 1 Translational and Clinical Research Institute, Newcastle University, Newcastle upon Tyne, UK; 2 Paediatric Haematopoietic Stem Cell Transplant Unit, Great North Children’s Hospital, Clinical Resource Building, Floor 4, Block 2, Queen Victoria Road, Newcastle upon Tyne, NE1 4LP UK; 3 Great Ormond Street Hospital NHS Trust, London, UK; 4 Immunodeficiency Centre for Wales, University Hospital of Wales, Cardiff, UK; 5 Division of Infection & Immunity, School of Medicine, Cardiff University, Cardiff, UK; 6 Department of Pediatrics, Faculty of Medicine, Kuwait University, Kuwait City, Kuwait; 7 Department of Clinical Immunology, Addenbrooke’s Hospital, Cambridge, UK; 8 Laboratorio de Inmunologia, UNAM, FES Cuautitlan, Cuautitlan, Mexico; 9 University College London NHS Foundation’s Trust/University College, London, UK; 10 Children’s Hospital Iceland, Landspitali - University Hospital, Reykjavík, Iceland; 11 Division of Allergy and Immunology, The Children’s Hospital of Philadelphia, Philadelphia, PA USA; 12 Center for Chronic Immunodeficiency, Medical Center, Faculty of Medicine, Albert-Ludwigs-University, Freiburg, Germany

**Keywords:** Autosomal dominant hyper IgE syndrome, dominant-negative STAT3 mutations, hematopoietic stem cell transplantation, Job syndrome, STAT3-HIES T_H_17 cells

## Abstract

Autosomal dominant hyper-IgE syndrome caused by dominant-negative loss-of-function mutations in signal transducer and activator of transcription factor 3 (*STAT3*) (STAT3-HIES) is a rare primary immunodeficiency with multisystem pathology. The quality of life in patients with STAT3-HIES is determined by not only the progressive, life-limiting pulmonary disease, but also significant skin disease including recurrent infections and abscesses requiring surgery. Our early report indicated that hematopoietic stem cell transplantation might not be effective in patients with STAT3-HIES, although a few subsequent reports have reported successful outcomes. We update on progress of our patient now with over 18 years of follow-up and report on an additional seven cases, all of whom have survived despite demonstrating significant disease-related pathology prior to transplant. We conclude that effective cure of the immunological aspects of the disease and stabilization of even severe lung involvement may be achieved by allogeneic hematopoietic stem cell transplantation. Recurrent skin infections and abscesses may be abolished. Donor T_H_17 cells may produce comparable levels of IL17A to healthy controls. The future challenge will be to determine which patients should best be offered this treatment and at what point in their disease history.

## Introduction

Autosomal dominant hyper-IgE syndrome is a rare primary immunodeficiency (PID) with multisystem pathology [1], caused by dominant-negative loss-of-function (LOF) mutations in signal transducer and activator of transcription factor 3 (*STAT3*) [2, 3] (STAT3-HIES). STAT3 is integral to lymphocyte development and differentiation. STAT3-deficient humans and mice have impaired cellular and humoral immune responses [4, 5], explaining the diverse immunologic manifestations [2–4]. In the context of infection, there is poor differentiation of T lymphocytes into T_H_17 cells, resulting in low IL17 and IL22 production [6], which contributes to susceptibility to encapsulated organisms and fungi.

HSCT was initially reported as unsuccessful in STAT3-HIES by our group [7]. Based on this impression, supportive treatment has been largely adopted [1], but many patients develop severe life-limiting pulmonary disease. However, over the years, eight case reports of allogeneic hematopoietic stem cell transplant (HSCT) for STAT3-HIES have been published [7–12], three of them performed following the diagnosis of non-Hodgkin’s B cell lymphoma, which is a known complication of this primary immunodeficiency [8]. In these eight cases, five patients had received myeloablative conditioning regimens, while three had reduced-intensity conditioning. Seven patients, including two with Hodgkin’s lymphoma, were recently reported as alive and well 4–20 years following report of HSCT [13], while one patient had died from the pre-existing lymphoma post-transplant [10].

We initially reported failure of HSCT to resolve or cure the immune defect in our first patient. However, we now acknowledge that STAT3-HIES patients have experienced sustained benefit from HSCT. At the time of our report, the molecular cause of STAT3-HIES was unknown, and we based our statements on failure to normalize the serum IgE level and an apparent failure to change the immunological defect. We have now shown that the IL17A levels of that patient approaches normal (although not measured pre-transplant) and memory B-lymphocytes are present. We now report a series of eight patients successfully treated with HSCT in the UK, including the long-term follow up of our previously reported patient [7].

## Methods

We conducted a retrospective review of medical records of STAT3-HIES patients from two UK transplant centers for PID. We included only patients with confirmed dominant-negative *STAT3* mutations who have undergone allogeneic HSCT. We recorded pre-transplant co-morbidities, conditioning, outcome, and on-going co-morbidities in eight patients who received nine transplants.

In selected patients for whom samples were available, IL17A levels were measured by Luminex (Bio-Plex, Biorad, UK) [14], 24 hours after stimulation in whole blood (96 Costar-F plates, 1:5 diluted in RPMI) of healthy controls and patients. Stimulations were performed using PHA (Sigma-Aldrich, 10 μg/ml) and compared with unstimulated medium samples. Data were compared with historic controls, which had been stimulated under identical conditions. *P* values were calculated using the two-tailed Mann-Whitney test or the Wilcoxon matched pairs test, excluding the data of P5 before HSCT.

Parents, and, where appropriate, patients consented to the procedure and data collection.

## Results

Transplants were performed between 1996 and 2019. Demographic data, genetic mutation, and pre-HSCT clinical status are summarized in Table 1. All patients had dominant loss-of-function mutations in *STAT3* with a classical clinical phenotype, and all had significant lung or skin-related complications prior to HSCT.

**Table 1:** Patient demographic features and pre- and post-HSCT clinical details

Patient no.; sex	Age at HSCT (years)	STAT3 Heterozygous mutation	Skin disease		Lung disease and PFTs		Fractures; other skeletal disease		Infection; other Issues	
			Pre-HSCT	Post-HSCT	Pre-HSCT	Post-HSCT	Pre-HSCT	Post-HSCT	Pre-HSCT	Post-HSCT
1 M	7	c.1909 G>A p.V637M	Yes	Normal	Left upper and lower lobectomies with bronchopleural fistula; bilateral bronchiectasis; chronic *Aspergillus* infection; pulmonary hypertension FEV1 47% FVC 51%	Stable appearance on CT: bronchiectasis persist Nocturnal CPAP for lobar collapse post-scoliosis repair with limited exercise tolerance FEV1 20% FVC 23%	Yes	2 further fractures at 3–4 years post-HSCT Development of scoliosis requiring spinal fusion	*Staphylococcus aureus* pericarditis	-
2 F	6	c.1771 A>G p.K591E	Yes	Normal	Recurrent *Pseudomonas* lung infection with cyst formation Not done	Stable; no progression of pulmonary cysts FEV1 95% FVC 88%	No	Development of scoliosis	-	Gonadal failure following myeloablative conditioning Arterial hypertension
3 M	13	c.1144 C>T p.R382W	Yes	Normal	Severe and recurrent pneumonia with bronchiectasis FEV1 63% FVC 70%	Stable appearance on CT No further hemoptysis FEV1 83% FVC 71%	Yes	No	-	ST segment elevation myocardial infarction aged 26 years (13 years post-HSCT)
4 M	14	c.1144 C>T p.R382W	Yes Pustular dermatitis	Dry skin, no infection	Bronchiectasis with multi-cystic lung disease, leading to left lobectomy FEV1 28% FVC 26%	Significant improvement in pulmonary cystic disease and resolution in right lower lobe bronchial thickening on CT FEV1 65% FVC 74%	Yes	No	*Staphylococcus aureus* liver abscess	Autoimmune neutropenia
5 F	17	c.1144 C>T p.R382W	Yes Persistent infected czema	Normal	Pneumatocoele leading to left lobectomy FEV1 53% FVC 67%	Improvement in lung appearance on CT FEV1 51% FVC 67%	Yes	No	-	Aspergillus perdicarditis at 7 months post-HSCT resolved by 16 months post-HSCT
6 M	18	c.1110 A>G D371_G380del	Yes Eczema, pustular dermatitis	Resolution of previous chronic infection	Lung abscess; pulmonary TB FEV1 89% FVC 90%	Stable CXR changes Not done	No Retained primary dentition	Enterococcal septic arthritis with collapse of femoral head	Recurrent *Staphylococcus aureus* liver abscess Candida retroperitoneal lymphadenitis and liver abscess	Early gastrointestinal bleed in post-transplant period Medication-related nephrotoxicity
6 2^nd^ HSCT	18									
7 M	13	c.1144 C>G p.R382G	Yes Multiple skin abscesses	Dry skin, no infection	Minimal FEV1 89% FVC 90%	Improvement in symptoms and exercise tolerance Not done	No Retained primary dentition	No	Osteomyelitis of mandible	-
8 F	6	c.1144 C>T p.R382W	Yes Eczema, Multiple skin abscesses	Dry skin, no infection	Severe bronchiectasis bilaterally with broncho-pulmonary aspergillosis FEV1 84% FVC 92%	Improvement in symptoms FEV1 82.7%FVC 78.4%	No	No	-	-

Details of conditioning regimen, cell source, and graft-versus-host disease (GvHD) prophylaxis are detailed in Table 2. One patient received conventional myeloablative conditioning, four received reduced-toxicity myeloablative conditioning, and three received reduced intensity conditioning, one of whom rejected and was successfully re-transplanted following a reduced-toxicity myeloablative regimen (patient six). All inoculi were matched at 10/10 HLA-loci, apart from the first product for patient six, which was a 9/10 match (DQ mismatch).

**Table 2:** Patient transplant demographics

Patient	Follow-up	Cell source; HLA Match	Conditioning regimen	GvHD prophylaxis	Acute GvHD	Latest donor chimerism
1	6 years	URD PBSC 10/10	Alemtuzumab 1mg/kg Fludarabine 150mg/m^2^ Melphalan 140mg/m^2^	CSA MMF	-	CD15 55% CD19 55% CD3 86%
2	20 years	URD BM 10/10	Alemtuzumab 1mg/kg busulphan 16mg/kg Cyclophosphamide 200mg/kg	CSA	Grade 1 skin	WB 100%
3	11 years	URD BM 10/10	Alemtuzumab 1mg/kg Fludarabine 150mg/m^2^ Melphalan 140mg/kg	CSA MMF	-	WB 100%
4	3 years	MSD PBSC 10/10	Alemtuzumab 1mg/kg Treosulfan 42g/m^2^ Fludarabine150mg/m^2^	CSA MMF	Grade 1 skin	CD15 100% CD19 100% CD3 91%
5	3 years	URD PBSC 10/10	Alemtuzumab 1mg/kg Fludarabine 150mg/m^2^ Treosulphan 42g/m^2^	CSA MMF	Grade 1 skin	WB 100%
6	-	Mismatched URD PBSC 9/10 (DQ mismatch)	Alemtuzumab 60mg Fludarabine 150mg/m^2^ Melphalan 140mg/m^2^	CSA	-	Hyperacute rejection D+13
	2 years	URD BM 10/10	ATG Fludarabine 150mg/m^2^ Treosulphan 42g/m^2^ Thiotepa 10mg/kg	CSA MMF	-	WB 100%
7	20 months	URD PBSC 10/10	Alemtuzumab 1mg/kg Fludarabine 150mg/m2 Treosulphan 42g/m2	CSA MMF	-	WB 100%
8	15 months	MSD BM 10/10	Alemtuzumab 1mg/kg Fludarabine 150mg/m^2^ Treosulphan 42g/m^2^	CSA MMF	-	CD15 96% CD19 94% CD3 94%

All patients survived and no patient experienced significant GvHD. Seven patients had complete or high level (>90%) donor chimerism and one had partial chimerism; however, all had improvement in immune phenotype, with a reduction in clinical symptoms, halting of progression of lung disease and improvement in skin infections. Non-immune manifestations showed variable improvement, with one patient developing further fractures and two patients demonstrating worsening scoliosis post-transplant. One patient has recently had vasculopathy with an ST segment elevation myocardial infarction with ectatic coronary vasculature [12]. Patient clinical status post-HSCT are summarized in Table 3.

**Table 3:** Patient immunological data pre- and post-HSCT

Patient	IgE (kU/L)		IVIG given		Vaccine responses; CSM/Mem B lymphocytes (25–75th centile reference range)		Antimicrobial prophylaxis post-HSCT
	Pre-HSCT	Post-HSCT	Pre-HSCT	Post-HSCT	Pre-HSCT	Post-HSCT	
1	4007	1720	Yes	No	Normal tetanus, absent HiB and pneumococcus -	Normal -	Voriconazole; azithromycin
2	75,000	2641	Yes	No	Normal -	Normal CSM B 9% (9.2–18.9%) Mem B 13% (13.4–21.4%)	Azithromycin
3	>6000	176	Yes	No	Normal -	Normal -	Doxycycline
4	81,097	11,813	Yes	No	Normal CSM B 1% (3.3–9.6%) Mem B 3% (4.6–10.2%)	Normal CSM B 1% (3.3–9.6%) Mem B 3% (4.6–10.2%)	Azithromycin
5	4487	1939	No	No	Normal CSM B 1% (3.3–9.6%) Mem B 3% (4.6–10.2%)	Normal CSM B 2% (7.2–12.7%) Mem B 7% (7.4–13.9%)	Posaconazole; nebulized amphotericin B; azithromycin
6	143,000	-	Yes	No	Normal CSM B 0.4% (3.3–9.6%)	- -	-
7	>5000	1636	Yes	No	Normal -	In progress Mem B cells 1.6% (3.3–9.6%)	-
8	1880–11,756	392	Yes	Yes	Normal tetanus and HiB, absent PPSV23 CSM B 2.6% (5.2–12.1%)	(On Ig) CSM B 3% (5.2–12.1%) Mem B 2% (7.5–12.4%)	Azithromycin

Reference ranges of 25–75th centile for CSM and Mem B lymphocytes from Morbach et al. [15]

*CSM* class switched memory, *HSCT* hematopoietic stem cell transplantation

**Fig. 1:**
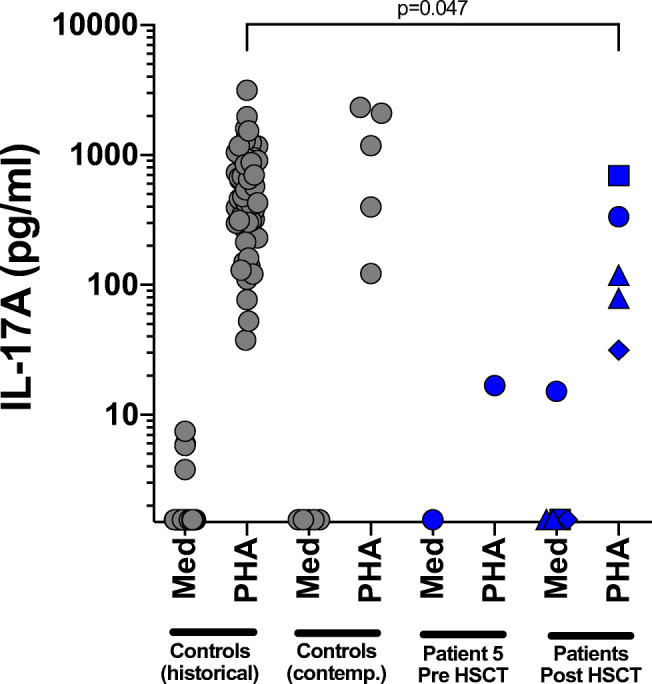
IL17A levels in healthy controls and patients post-transplant. IL17A levels were measured by Luminex (Bio-Plex, Biorad, UK) 24 hours after stimulation in whole blood (96 Costar-F plates, 1:5 diluted in RPMI) of healthy controls (historical and contemporary) and patients (P2—blue diamonds, P3—blue triangles, P4—blue squares, P5 (pre and post HSCT) —blue circles). Stimulations were done using PHA (Sigma-Aldrich, 10 μg/ml) and compared with unstimulated medium samples (Med)

In the one patient (patient 5) for whom pre- and post-transplant IL17A levels were available, IL17A was higher post-transplant. For patients 2, 3, and 4, IL17A levels were present post-transplant, but lower than controls (Fig. 1).

Patient 1 had significant pulmonary infection (complicated cystic bronchiectasis with *Pseudomonas* infection and widespread *Aspergillus* infection leading to pulmonary hypertension) with two lobectomies, complicated by development of bronchopleural fistula. He subsequently developed a *Staphylococcus aureus* pericarditis requiring surgical drainage, complicated by prolonged air leak. Transplantation at age 7 years was relatively uncomplicated. There was no viral re-activation, progression of chest lesions, or acute graft versus host disease. Six years post-transplant, his eczema had resolved and he was no longer getting significant respiratory infections. Immunoglobulin support has ceased, and normal vaccine and T-lymphocyte proliferative responses have been demonstrated. His scoliosis, exacerbated by his two previous left sided lobectomies, had deteriorated requiring spinal fusion surgery. Following this, his right lower airway integrity has been compromised, severely impairing his respiratory capacity. He now is reliant on overnight CPAP and can only ambulate short distances.

Patient 2 experienced frequent staphylococcal skin infections and cystic bronchiectasis infected with *Pseudomonas*, because of which transplantation was offered when aged 6 years. Despite 100% donor chimerism, the procedure was initially described as unsuccessful [7] because of continued elevated serum IgE. However, long-term follow-up confirmed sustained clinical improvement with no further respiratory or skin infections. Serum IgE levels diminished and remained reduced, and immunoglobulin support is no longer required. She developed thoracolumbar scoliosis, arterial hypertension, and gonadal failure. With 20 years follow-up, pulmonary status includes stable lingular bronchiectasis and non-progressive right upper lobe pulmonary cysts.

**Fig. 2:**
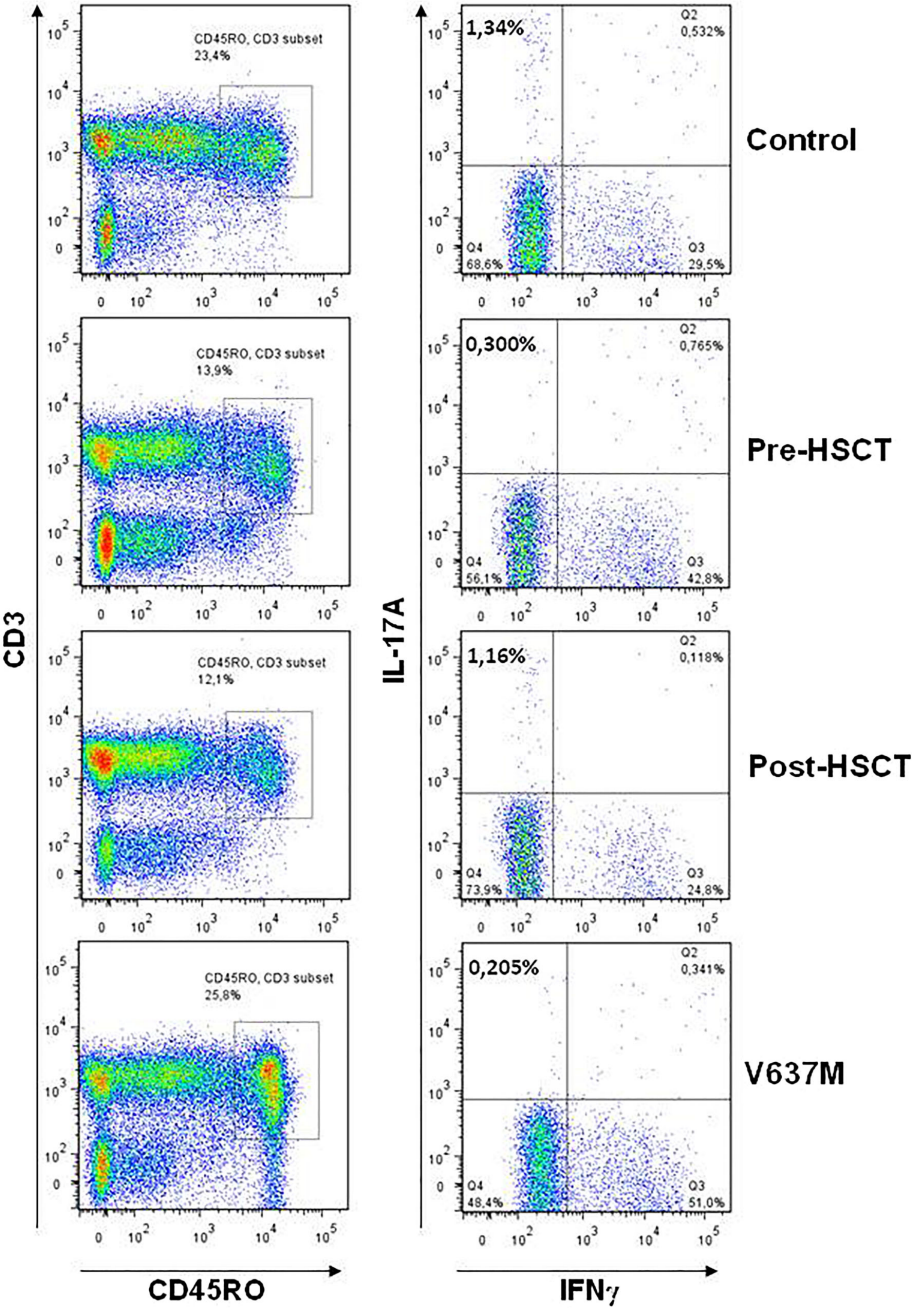
Pre- and post-transplant peripheral blood mononuclear cells demonstrate that IL17A is absent pre-transplant and present post-transplant (patient 3), with normal control and a negative control patient who has not been transplanted (p.V637M)

Patient 3 experienced frequent severe bronchopneumonias and progressive bronchiectasis despite immunoglobulin replacement, as well as osteopenia and lumbar vertebrae compression fractures. Transplantation at 13 years was relatively uneventful, and 13 years post-transplant, he demonstrates 100% donor chimerism with reduced serum IgE levels. Lung function has improved to normal values. Hemoptysis has stopped, and there is no computerized tomographic evidence of pulmonary disease progression. An increase in IL17A production after polyclonal stimulation was demonstrated post-HSCT (Fig. 2), and T_H_17 cells display normal response to IFN-γ and IL12 and normal IL12 production (data not shown). However, at 11 years post-HSCT, he sustained an anterior myocardial infarction, accompanied by classical clinical, electrocardiographic and biochemical signs and parameters. Angiography revealed proximal ectasia causing mid-vessel occlusion of his left anterior descending coronary artery but otherwise unobstructed coronary vessels. He was commenced on aspirin 75 mg for 3 months with rivaroxaban 2.5 mg BID for 3 years and clopidogrel 75 mg long term [12].

Patient 4 developed bronchiectasis, *Staphylococcus aureus* liver abscess, and recurrent skin abscesses. Severe multi-cystic and suppurative lung disease warranted left upper lobectomy. Following transplantation at age 14 years, repeat computerized tomography demonstrated significant widespread decrease in the number and size of previously reported large cysts, and there was a clinical improvement in the eczematous skin lesions. Post-transplant IL17A production was normal.

Patient 5 developed multiple abscesses and pneumatoceles during infancy leading to right upper lobectomy and broncho-pulmonary fistula at age 13 years. Pulmonary function was impaired prior to HSCT, aged 17 years. Non-invasive pulmonary *Aspergillus* infection persisted post-HSCT, treated with appropriate long-term anti-fungal therapy—at 7 months post-transplant, and Aspergillus pericarditis was diagnosed, which resolved with dual anti-fungal therapy by 16 months post-transplant. Thoracic computerized tomography demonstrated a dramatic improvement in lung appearance, and the residual right upper lobe cavity remained stable.

Patient 6 had persistent multiple skin infections with severe eczema and lymphadenitis through childhood and a single lung abscess. Additionally, a persistent *Staphylococcus aureus* hepatic abscess required surgical drainage on four occasions. Further to this, the patient required surgical excision of *Candida albicans* retroperitoneal lymphadenitis and a fungal liver abscess*.* The patient developed bowel perforation of unknown etiology at 6 years of age, before HSCT. An immune-mediated acute rejection of first graft was confirmed at day 13 post-transplant, followed by an autologous recovery. In this period, the patient experienced an enterococcal septic arthritis, with collapse of femoral head, gastrointestinal hemorrhage, and further episodes of fungal lymphadenitis. A second transplant procedure using a reduced toxicity treosulfan-based regimen was successful. The patient has good immune reconstitution and has ceased immunosuppression and immunoglobulin replacement.

Patient 7 was first referred to pediatric care at the age of 4 weeks due of eczema and skin infections, mainly on the face and scalp, from which *Staphylococcus aureus* was isolated and treated. Serum IgE levels were elevated prompting a diagnosis of Hyper IgE syndrome, confirmed genetically. The patient continued to have numerous recurrent skin abscesses, requiring drainage over 20 times per year despite antibiotic treatment and immunoglobulin replacement. He subsequently developed mandibular osteomyelitis. Pulmonary disease was limited to asthma only, with no significant infection. Transplant was uncomplicated, and at 20 months post-HSCT, his skin is much improved with no new infections. He has discontinued immunoglobulin and is awaiting vaccination.

Patient 8 presented as a neonate with staphylococcal skin infection and went on to develop recurrent skin infection, pneumonia, and otitis media. She demonstrated elevated serum IgE with poor pneumococcal antibody titers following vaccination and almost no T_H_17 cells. Her pulmonary disease progressed with findings of *Aspergillus* on bronchoalveolar lavage leading to a diagnosis of allergic bronchopulmonary aspergillosis, treated with antifungal therapy, corticosteroids, and anti-IgE monoclonal antibodies. She underwent HSCT aged 6 years with an uncomplicated course. One year post-HSCT, she reports only dry skin with no infection. Pulmonary function is stable and she is asymptomatic from cough or dyspnoea. Serum IgE has fallen to 392 kU/L. She remains on immunoglobulin replacement but has normal B lymphocyte numbers.

## Discussion

Published data on the outcome of allogeneic HSCT for STAT3-HIES patients are limited, perhaps stemming from our initial report indicating non-utility [7]. A recent report by Oikonomopoulou et al. [13] summarizes the data of 7 patients transplanted between 1998 and 2018, one of which was the patient we originally reported in 2000 [7]. For three of these, the transplant indication was lymphoma [9, 10], and thus, although the STAT3-HIES was reported as cured, their transplant indication and management could be considered separately. The first of these patients died 6 months following transplant from interstitial pulmonary fibrosis, although with pre-existing abnormal immune parameters, including raised IgE, normalized post-transplant [10]. The other two patients, with follow up of 10 and 14 years, respectively, both with 100% donor chimerism, were reported to have normalized immunological parameters including a normalized proportion of T_H_17^+^ lymphocytes. Furthermore, other abnormalities associated with STAT3-HIES, including coarse facies and osteoporosis, were reported to have resolved, and there was no symptomatic development of STAT3-HIES-associated vasculopathy, although it is not clear if any screening was performed [9]. A further patient, transplanted because of a complicated infectious history, was reported after 42 months of follow-up. The patient had full donor chimerism and remained infection-free after discontinuing anti-microbial prophylaxis. Vaccine antibody responses were demonstrated and IgE normalized, STAT3 signaling was restored in hematopoietic-derived cells, and the percentage of Th17^+^ lymphocytes and central memory T lymphocytes to normal ranges was established [11]. Finally, 2 patients, transplanted for recurrent infection, were reported with 8 and 10 years of follow-up, respectively. Immunological parameters normalized and the infection frequency significantly diminished, but non-immunological features of STAT3-HIES remained, including a propensity to recurrent fractures and development of a new pneumatocele, associated with ≤50% donor chimerism [8].

**Fig. 3:**
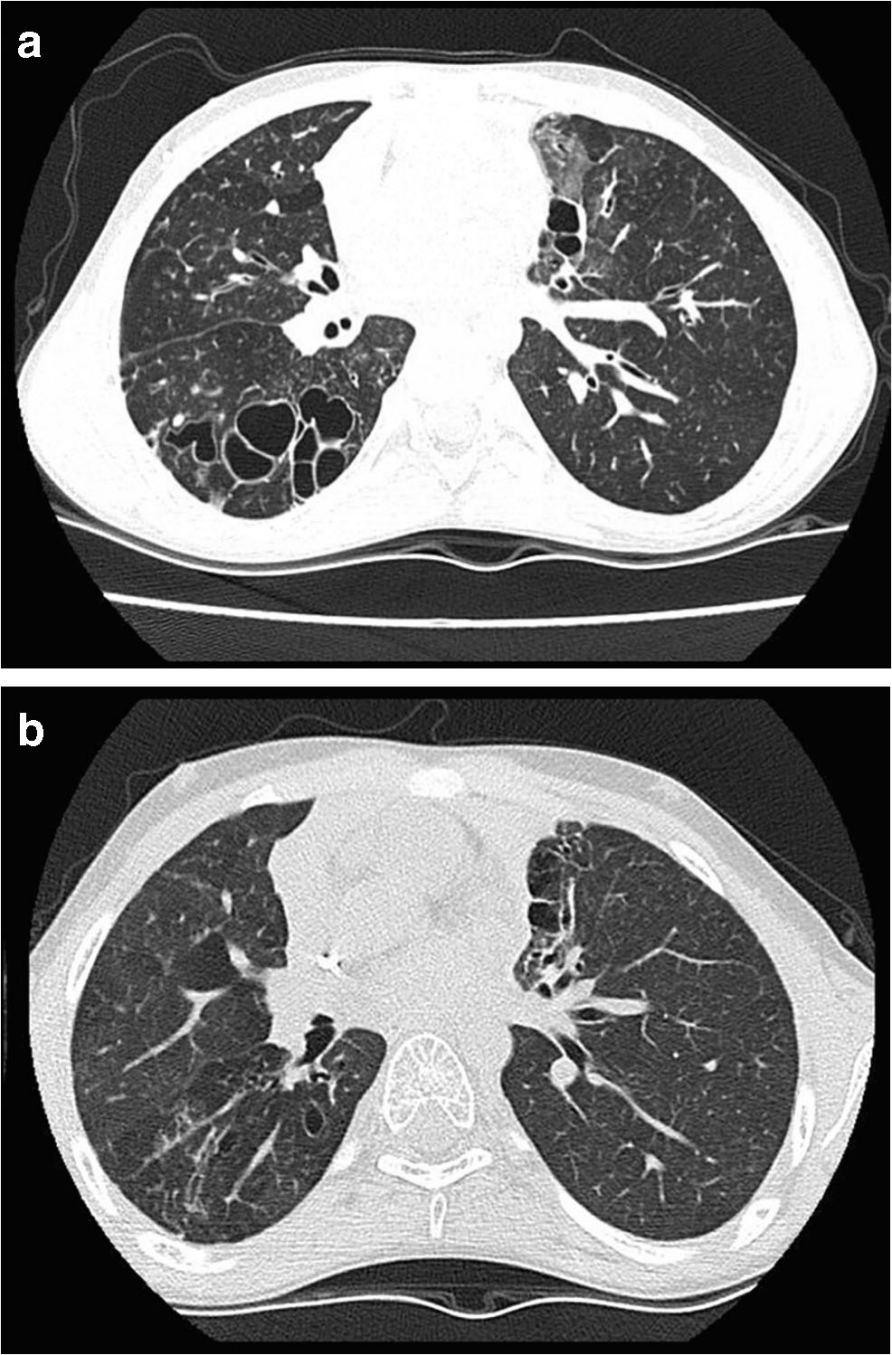
Thoracic computerized tomography images from patient 4. **a** Images taken 1 year pre-transplant showing markedly abnormal lung parenchyma with large cysts, bullae, and cystic bronchiectasis particularly in the right lower lobe in association with cylindrical bronchiectasis, bronchial wall thickening, bronchocoeles, and reduced lung attenuation. **b** Images taken 1 year post-transplant showing several thin walled pulmonary “cysts,” similar to previous pre-transplant findings and are therefore most likely to result from previous infection and lung destruction. Some regions of parenchymal distortion and scarring are also similar. There is some bronchial dilatation and distortion in regions of scarring, but no convincing evidence of bronchiectasis

Our current report extends follow-up of the initial patient to over 18 years and reports on a further 7 patients, one recently published in the context of new onset vasculopathy complications [12], thus contributing the largest series to date. Perhaps the most remarkable observation is that despite pre-transplant severe, progressive lung disease—itself a significant risk factor for HSCT outcome—as well as other significant pre-transplant sequelae, all patients in our series survived. Importantly, pulmonary disease has stabilized in all and improved in some, as demonstrated on clinical, functional, and radiological parameters (Table 1; Fig. 3). No peri-transplant pulmonary inflammatory complications were observed, unlike the post-HSCT course seen in many PID patients. GvHD was infrequent and insignificant. Notably, no patients have required further lung surgery post-HSCT. Most patients have remained on some antimicrobial prophylaxis, in view of treating physician concerns of recurrent infections in pre-existing bronchiectatic lungs—despite this, control of lung disease reported by patients has stabilized or improved. Aspergillus infection, likely from pre-existing colonization or infection, recurred through, or after transplantation in some, but did not cause the severe complications usually associated with transplant-associated aspergillosis. Other significant infections reduced in frequency or resolved completely, accepting that most patients remained on antimicrobial prophylaxis. Importantly, eczema improved in all patients and skin infections were abolished post-transplant.

STAT3 plays an important part in normal endothelial cell biology [16], and endothelial dysfunction is known to contribute to initiation of transplant-related inflammatory complications [17]. It may be that the lack of severe post-HSCT inflammatory complications including acute graft-versus-host disease, observed in these patients and the previously reported cases, might be due to reduced endothelial cell upregulation, conferred by dominant negative STAT3-LOF mutations.

Although serum IgE levels remain above the adult reference range, they are substantially lower post-HSCT compared with pre-HSCT levels, although, in non-transplanted patients, IgE levels may fall over time [1]. More importantly, in those patients for whom data were available, normal T_H_17 function and associated cytokine responses are demonstrated (Fig. 1), confirming previous reports [8, 9, 11].

As expected, many extra-immune manifestations have not resolved post-HSCT, and patients retain coarse facial features, as well as bone-related complications, although it is noteworthy that only one patient has developed fractures post-transplantation. No patient has developed lymphoma to date. Critically, however, one patient (#3) recently experienced an anterior myocardial infarction, with evidence of thrombotic occlusion of dilated (ectatic) coronary vessels upon angiography. We estimated this patient’s 10-year risk of developing a heart attack or stroke, using the QRISK3 calculator, as 0.1%, which suggests that conventional cardiovascular risk factors were unlikely to account for the event, and disease-associated vasculopathy is more likely to be implicated [12]. Screening of coronary vasculature had not been performed prior to this event, and so it is unclear whether HSCT ameliorated, accelerated, or had no effect on these coronary vessels. Whether earlier HSCT will favorably alter the pre-disposition to vascular complications documented in these patients remains to be seen. Further research is required to establish the clinical significance of these wider complications of STAT3-HIES and capture the long-term impact of STAT3-HIES on patients’ quality of life across the spectrum of presentations.

Contrary to our previous assertion [7], this series, along with other published reports, further supports the notion that allogeneic HSCT can improve the immunological deficit, modify the pulmonary course in these patients, and improve the skin condition. The future challenge will be to identify which patients will benefit from early consideration of this therapy to prevent end-organ damage, reduce hospitalization and improve quality of life, as well as which conditioning regimen to use. The importance of complete or high donor chimerism is not established, and further work is required to determine optimum conditioning regimens and minimum effective donor chimerism level. Furthermore, it will be particularly important to determine whether HSCT can alter the risk of developing vascular anomalies, which may be associated with significant morbidity and mortality.

## Data Availability

For further information please contact ARG: a.r.gennery@ncl.ac.uk.
